# Imaging features of bronchogenic cyst of the stomach: A case report with literature review

**DOI:** 10.1097/MD.0000000000041338

**Published:** 2025-01-31

**Authors:** Deshuo Dong, Anliang Chen, Ailian Liu

**Affiliations:** a Department of Radiology, The First Affiliated Hospital of Dalian Medical University, Dalian, Liaoning, P.R. China; b Dalian Medical Imaging Artificial Intelligence Engineering Technology Research Center, Dalian, Liaoning, P.R. China.

**Keywords:** case report, gastric bronchogenic cyst, preoperative diagnosis

## Abstract

**Rationale:**

Bronchogenic cyst (BC) is a congenital disease characterized by an anomaly of the foregut in the embryonic stage. Gastric BC is exceedingly rare and has never been accurately diagnosed prior to surgery; it is often misidentified as gastric stromal tumor, with the definitive diagnosis confirmed through postoperative specimens. Although gastric BC is considered a benign lesion, its prognosis remains uncertain, underscoring the importance of accurate preoperative identification.

**Patient concerns:**

The present study reported the case of a 64-year-old female who presented with 2 incidentally detected lesions of the gastric corpus and antrum. Computed tomography and magnetic resonance imaging showed cystic lesions with delayed enhancement of the cyst wall and no enhancement of the cyst contents.

**Interventions and Diagnoses:**

The patient underwent a laparoscopic partial gastrectomy. BC was diagnosed by histopathology and immunohistochemistry after surgery.

**Outcomes:**

The patient had an uneventful hospital course and was discharged on the eleventh postoperative day. No recurrence or metastasis was observed after 33 months.

**Lessons:**

BC of the stomach is mostly ovate in shape with well-defined margins. The cyst wall shows prolonged enhancement and calcification may occur at the edges. The density and signal of the cyst content varied with composition. These imaging features are helpful for differentiating diagnoses from other diseases.

## 1. Introduction

Bronchogenic cysts (BCs) are rare congenital cystic lesions caused by congenital dysplasia of the respiratory system during fetal life from weeks 3rd to 7th weeks,^[1]^ with a prevalence rate of 1/68,000 to 1/42,000.^[2]^ BCs in the stomach is extremely rare, and only a few cases have been reported. Due to the lack of specific clinical and radiological features, they were not correctly diagnosed preoperatively. In this study, we present a case of gastric BC with 2 lesions, in which both computed tomography (CT) and magnetic resonance imaging (MRI) were performed, and a systematic literature review was conducted. We highlighted the key imaging features in the diagnosis of gastric BC to improve our understanding of this disease. We present the following case in accordance with the CARE-Checklist.

## 2. Case presentation

A 64-year-old woman was admitted to our hospital due to a “stomach mass” detected incidentally upon a routine health examination 2 months ago. Abdominal ultrasound was performed at another hospital, which revealed the possibility of gastric space-occupying lesions. The patient denied abdominal discomfort, fever, nausea, vomiting, belching, hematemesis, or weight loss. She had a history of hypertension and atrial fibrillation. She took medication regularly, and her symptoms and blood pressure were well-controlled. The physical examination findings were unremarkable. The patient had no family history of gastric cancer. Routine blood examination, coagulation, liver and renal function tests, and tumor markers, including AFP, CEA, CA125, CA19-9, and CA72-4, were within normal limits, and a fecal occult blood test was negative.

**Figure 1.**
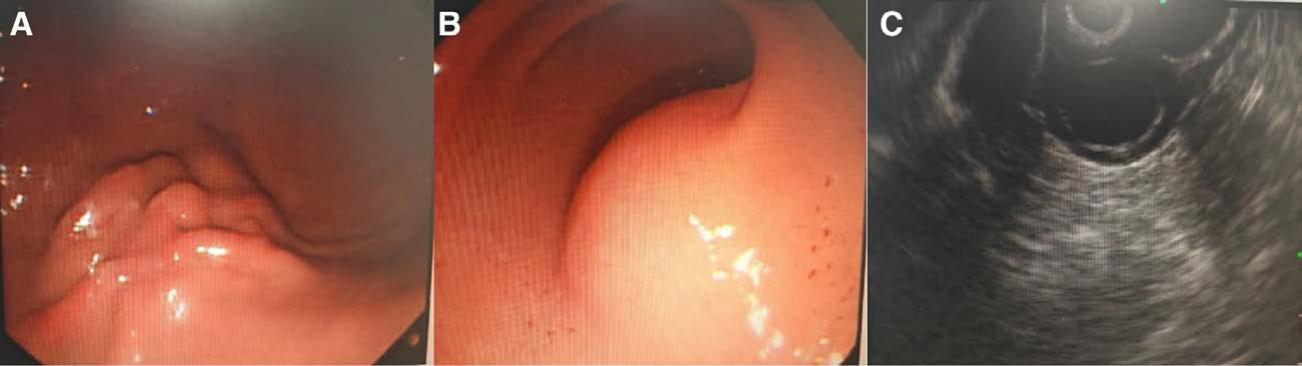
(A and B) represented gastroscopic images, (A) showed a submucosal bulge in the distal part of gastric corpus of the greater curvature. The lesion was originated in the lamina musculi propria, showing heterogeneous hypoecho, with a size of 16 mm × 15 mm. The mucosa folds on the surface were immobilized and touched relatively hard. (B) showed another submucosal bulge in the posterior wall of gastric antrum of the greater curvature. The lesion was originated in the submucosa, showing homogeneity no echo with high echo envelope, with a size of 22 mm × 21 mm. The surface was smooth and touched soft. (C) represented ultrasonic gastroscopy image of the lesion in gastric antrum.

**Figure 2.**
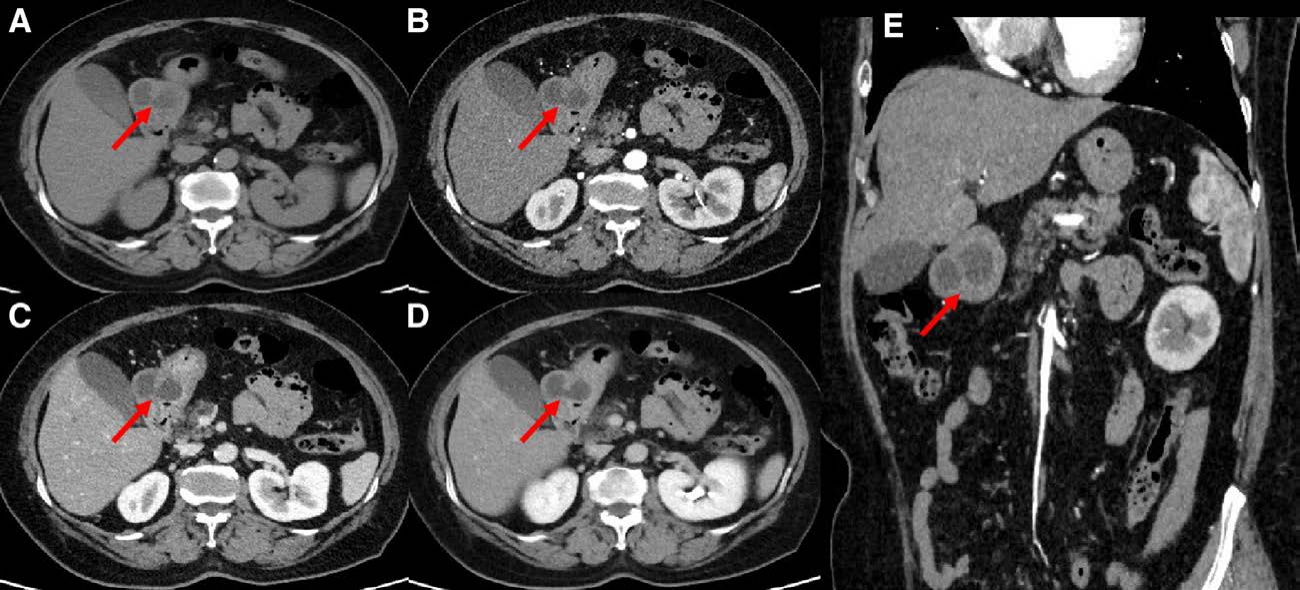
(A–E) represented axial CT plain scan, arterial phase, venous phase and delayed phase of enhancement scan, coronal arterial enhancement, respectively, showing the cystic lesion of gastric antrum with a larger axial cross-sectional area of 49 mm × 35 mm. The lesion was in the shape of ovate, with well-defined margin. The diameter of the thickest part of the cyst wall was about 9 mm, the CT values of plain and enhancement phase III were about 41HU, 45HU, 61HU and 70HU, respectively. The cyst contents showed low density and no enhancement, the CT values of plain and enhancement phase III were about 5HU, 6HU, 8HU, 9HU.

**Figure 3.**
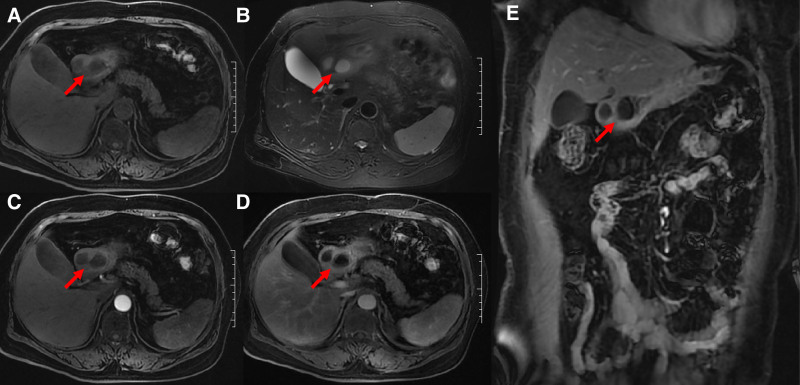
(A–E) represented T_1_WI, T_2_WI, axial early and delayed enhancement, coronal delayed enhancement, showing the same lesion as Figure 2. of gastric antrum. The cyst wall showed isointense on T_1_WI and T_2_WI, with prolonged enhancement, no wash-out until the delayed phase. The cyst contents showed hypointense on T_1_WI and hyperintense on T_2_WI, with no enhancement.

**Figure 4.**
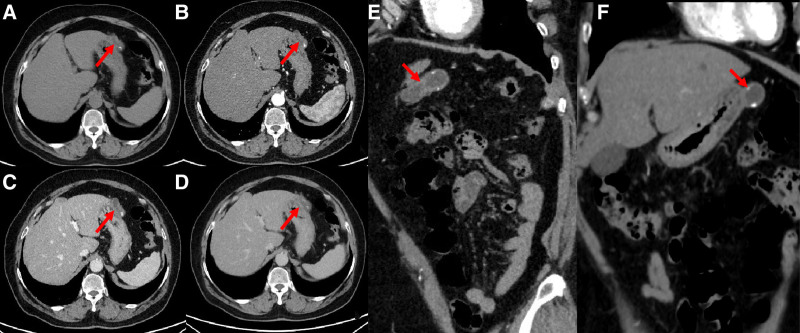
(A–D) represented axial CT plain scan, arterial phase, venous phase and delayed phase of enhancement scan, (E and F) represented oblique sagittal and coronal arterial enhancement, respectively, showing another cystic lesion protruding from gastric corpus of greater curvature, with a larger axial cross-sectional area of 36 mm × 17 mm. Punctate calcifications were found in the cyst wall. The CT features of cyst wall and cyst content were similar to the lesion of gastric antrum.

**Figure 5.**
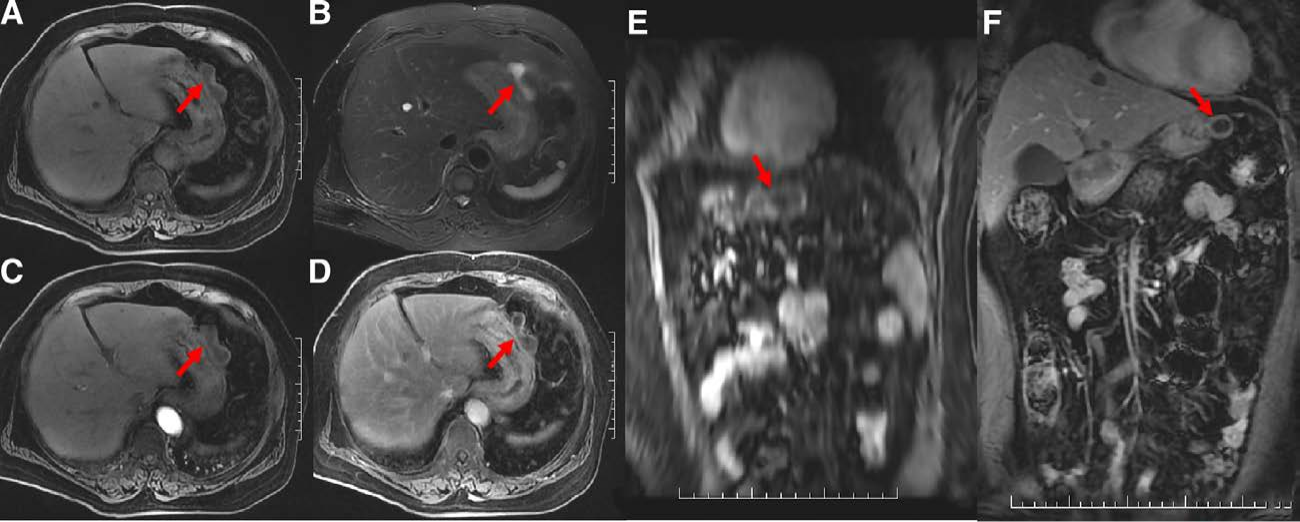
(A–D) represented T_1_WI, T_2_WI, axial early and delayed enhancement, (E–F) represented oblique sagittal and coronal delayed enhancement, respectively, showing the same lesion as Figure 4 of gastric corpus. The cyst wall showed isointense on T_1_WI and T_2_WI, with prolonged enhancement, the cyst contents showed hypointense on T_1_WI and hyperintense on T_2_WI, with no enhancement, similar to the lesion of gastric antrum.

After admission, the patient underwent gastroscopy and endoscopic ultrasonography, as was shown in Figure 1. Subsequently, CT and MRI were performed for detailed examination of the lesions, as shown in Figures 2–5. An abdominal enhanced CT scan was performed using GE Revolution CT (GE Healthcare, Milwaukee). Two lesions were observed in the greater curvature of the gastric corpus and antrum. Among the 2 lesions, the gastric corpus was exogenous, protruding outside the outline of the gastric wall, with a larger axial cross-sectional area of 36 mm × 17 mm, whereas another lesion of the gastric antrum was intermediate, protruding inward and laterally, with a larger axial cross-sectional area of 49 mm × 35 mm. Both lesions were ovate with well-defined margins and clear boundaries between the surrounding tissues. The lesions showed low internal density, and the CT values of plain and enhancement phase III were approximately 5HU, 6HU, 8HU, 9HU, with no reinforcement. Therefore, these 2 lesions were considered cystic. The diameter of the thickest part of the cyst wall was approximately 9 mm with a uniform density. The inner and outer surfaces were smooth, with no wall nodules or internal partitions. The plain CT value was approximately 41HU. After intravenous contrast medium injection, the cyst walls showed prolonged homogeneous enhancement, and the CT values of enhancement phase III were approximately 45HU, 61HU, and 70HU. Additionally, calcification was observed on the cyst wall of the lesion in the gastric corpus. No abnormally enlarged lymph nodes were observed around the stomach or the retroperitoneum.

The patient underwent 3.0T MRI (Signa Excite HDxt; General Electric Healthcare, Milwaukee). The lesions in the gastric corpus and antrum showed hypointensity on T_1_WI and hyperintensity on T_2_WI, with no enhancement after intravenous contrast medium injection, proving to be cystic lesions. The cyst walls were isointense on both T_1_WI and T_2_WI, with prolonged enhancement and no wash-out until the delayed phase, similar to CT findings.

**Figure 6.**
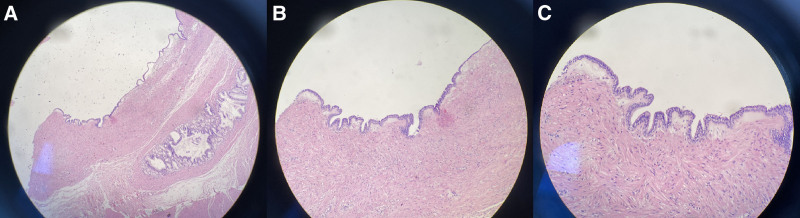
(A–C) represented pathological pictures under microscope of 40 times, 100 times and 200 times. Microscopic examination revealed a benign bronchogenic cyst, lined with pseudolayered columnar epithelium and single columnar/flat epithelium, no atypia was observed in the epithelium, and fibrous tissue and smooth muscle were found around the epithelium. (hematoxylin–eosin stain, ×40, ×100, ×20).

The patient underwent laparoscopic partial gastrectomy 1 week after the CT and MRI examinations. The lesions were located in the gastric corpus and antrum of the greater curvature; they were of the exogenous and intermediate types, with sizes of approximately 40 mm × 20 mm and 40 mm × 30 mm, respectively. Gross pathology revealed that the inner walls of the cystic lesions were smooth and contained mucoids. Microscopic pathology showed that the lesions were lined with pseudolayered columnar epithelium and a single columnar/flat epithelium; no atypia was observed in the epithelium, and fibrous tissue and smooth muscle were found around the epithelium, as shown in Figure 6. Immunohistochemical pathology showed muc-1(+), muc-2(−), muc4(+), muc-5AC(part+), muc5b(+), muc-6(part+), CDX −2(−), CK20(−), CK7 (+), Ki −67 (+3%), P16 (−), P53 (wild type), SATB2(−), TTF-1(+), vilin(part+). Based on the histopathological results, the final diagnosis was BCs of the stomach. The patient had an uneventful hospital course and was discharged on the eleventh postoperative day. No recurrence or metastasis was observed after 33 months.

## 3. Discussion

BC is a congenital abnormality of the tracheobronchial bud that arises from the outward protruding pouches of the primitive foregut. It is most frequently located in the mediastinum, pericardial areas, or pulmonary parenchyma depending on the level of abnormal budding that occurs during development. We report a case of BCs in the stomach that is extremely uncommon. The occurrence of BC in the abdominal cavity can be explained as follows. In the early embryonic stage, the thoracic and abdominal cavities are connected by a pericardial abdominal cavity tube, and the abdominal cavity is divided into 2 separate cavities by fusion of the pleura and peritoneum, which are components of the diaphragm. In BC, abnormal buds of the tracheobronchial tree are cut off and migrate to the abdomen.^[2]^

DEWING et al first reported an enterogenous cyst of the stomach wall in 1956,^[3]^ while Gensler presented a cyst lesion of the stomach, which was the only reported case lined with ciliated pseudostratified columnar epithelium.^[4]^ A systematic review of the PubMed database was performed using the following keywords: (BC[Title]) AND (stomach[Title]), which yielded 13 potentially relevant articles. The exclusion of articles without full text (n = 2, French) or not in English (n = 1, French) eventually resulted in a final count of 10 articles, among which 1 of the articles included 2 cases, describing 12 cases, including the current study, as shown in Table 1.^[1,2,5–12]^

**Table 1:** Summary of clinical and imaging features of gastric BCs in the literature.

First author (yr)	Age	Sex	Symptoms	Laboratory examination	Location	Number of lesions	Diameter (mm)	Shape	Margin	Calcification	CT	Enhancement	T1WI	T2WI	Treatment	Outcome	(Refs.)
Keohane (1988)	64	F	Nausea, vomiting, epigastric pain	Hemoglobin 10.4 g m%; elevated alkaline phosphatase count	Posterolateral corpus	Single	150	Multiloculated	NR	NR	NR	NR	NR	NR	Resection	NR	^[5]^
Song (2005)	62	F	–	–	Cardia/less	Single	15	Ovate	Well-defined	NR	Low-density	NR	NR	NR	Resection	NR	^[6]^
Shibahara (2009)	43	M	Epigastric pain	NR	Cardia/less	Single	90	Ovate	Well-defined	NR	NR	NR	NR	hyperintense	Resection	NR	^[7]^
Yang (2013)	50	M	–	NR	Fundus	Single	75	Ovate	Well-defined	NR	NR	NR	NR	NR	Resection	Alive/24 mo	^[2]^
Yang (2013)	37	F	–	–	Posterior	Single	100	NR	Well-defined	NR	Low-density	NR	NR	NR	Resection	NR	^[2]^
Chhaidar (2017)	65	F	Epigastric pain	–	Cardia	Single	80	Ovate	Well-defined	NR	NR	Heterogeneous	NR	NR	Resection	Alive/24 mo	^[8]^
Han (2019)	62	F	Abdominal pain	NR	Cardia/less	Single	64	Ovate	Well-defined	Nodule-shaped on the edge	32HU	–	NR	NR	Resection	NR	^[9]^
He (2020)	55	F	Epigastric pain	CA72-4 8.3IU/mL (postoperatively normal)	Fundus/less	Single	52	Quasi-circular	Well-defined	NR	29HU	NR	NR	NR	Resection	Alive/10 mo	^[10]^
Sun B (2020)	68	M	-	-	Fundus	Single	95	Ovate	Well-defined	Spot-shaped on the edge	17HU	–	NR	NR	Resection	Alive/12 mo	^[11]^
Kihara (2022)	30s	F	Epigastric pain	CA19-9 55.8U/mL (Postoperatively normal)	Less	Single	26	Ovate	Well-defined	NR	Low-density	Prolonged contrast effect at margin	Hypointense	Hyperintense	Resection	NR	^[12]^
Qian (2023)	45	F	Epigastric pain	–	Cardia	Single	30	Ovate	Well-defined	NR	NR	–	NR	NR	Resection	NR	^[1]^
Current study	64	F	–	–	Gastric body and antrum	Multiple	41 (the larger 1)	Ovate	Well-defined	Spot-shaped on the edge	10 HU	Prolonged contrast effect at margin	Hypointense	Hyperintense	Resection	Alive/33 mo	–

“NR” indicates not mentioned, and “–” indicates none.

According to previous cases reported in the above literature, BCs of the stomach occur in a wide range of ages (from 30s to 68 years); among them, females had a relatively large number, followed by males (9 females, 3 males). Regarding symptoms, the majority of the patients were occasionally found. Some patients show symptoms, including epigastric pain, abdominal distension, nausea, and vomiting, which may be related to enlarged cysts, secondary infections, perforations, or compression of adjacent structures.^[2]^ Older patients or those with a longer clinical history of symptoms tend to present with a larger mass and are more likely to have epigastric discomfort, gastric ulcer, gastroesophageal reflux, or occasionally cancer.^[13]^ With regard to laboratory examinations, most of the patients’ laboratory tests showed no abnormalities. Some patients’ CA72-4^[10]^ and CA19-9^[12]^ levels are slightly elevated before surgery and may return to normal after surgery. The elevated CA19-9 level was likely because the epithelial cells secrete CA19-9 due to its foregut origin, gradually accumulate in the cyst, and absorb it into the serum.^[14]^ Most gastric BCs are located in the upper portion of the stomach (fundus and cardia), mainly on the lesser curvature side, while the occurrence on the greater curvature side has rarely been reported. The lesions in the present case were atypical and were located in the greater curvature of the gastric corpus and antrum. As for the number of lesions, except for 2 lesions in the current study, all the above studies were single. The lesions ranged in diameter from 15 to 150 mm (CT/MRI measurement or pathological evaluation).

Gastroscopy is of limited value in the detection of gastric BCs. If the lesion protrudes inward and laterally from the gastric wall, its location and size may not be completely and accurately evaluated. This may explain why the lesion size measured using gastroscopy in this case was smaller and inconsistent with that measured using CT. Therefore, it is of great significance to explore noninvasive examination methods for CT and MRI.

This case report describes 2 BCs of the stomach for which both CT and MRI images are available. Combined with previous case reports, BCs of the stomach were mainly cystic on CT. The lesions were mostly ovate in shape, with homogeneous density and well-defined margins, occasionally leading to the compression of adjacent structures, which is consistent with the imaging characteristics of benign lesions. The cyst walls were usually thin and smooth, and sometimes partial thickness was observed, but there were no intra-cystic nodules. After the injection of the contrast agent, the cyst walls showed prolonged homogeneous enhancement. Spotted or nodular calcification of the cyst wall has been mentioned in some cases, including the current case, which may be a characteristic manifestation of this disease. Cyst fluid content affected the CT value, varying from as low as normal cysts to as high as solid tumors. In approximately half of the lesions, the cyst contents were of low density <20HU.^[10]^ In individual cases, some cysts may have equal or even slightly high density on plain CT scans because of the high protein content, hemorrhage, and infection in the cyst fluid, which can be easily misdiagnosed as gastrointestinal stromal tumors (GIST) or gastric leiomyomas (GLM).^[9]^ After the injection of the contrast agent, the cyst content showed no enhancement.

There are very few MRI features of gastric BCs. Martín^[15]^ reported cases of retroperitoneal BCs because of the presence of methemoglobin, mucin, or protein; cyst fluid usually showed iso-to hyperintensity on T_1_WI and did not decrease in the fat inhibitory sequence, consistent with the cases reported by Kurokawa^[16]^ and lou^[17]^ of gastric BCs. Based on this, Ubukata^[18]^ found MRI to be better than CT for identifying the contents of cystic lesions. In the present case, the cyst contents of the 2 lesions were hypointense on T_1_WI and hyperintense on T_2_WI, in accordance with cases reported by Ubukata^[18]^ and Kihara.^[12]^ The cyst walls were isointense on both T_1_WI and T_2_WI. After injection of the contrast agent, the cyst contents showed no enhancement and the cyst walls showed prolonged homogeneous enhancement, similar to that observed on CT.

BCs of the stomach should be distinguished from the following diseases. On the 1 hand, BCs with low density on CT should be differentiated from cystic lesions, including foregut cysts and gastric duplication cysts, which are differentiated from BCs by the type of lining epithelium and surrounding layer. BC is traditionally used to describe cysts lined by pseudostratified columnar or cuboidal ciliated (respiratory) epithelium (PCCE) in the presence of cartilage or glandular tissue in the cyst wall.^[8]^ Lymphangioma: Abdominal cystic lymphangiomas often develop in the abdominal spaces. CT revealed a well-defined thin-walled cystic lesion with multilocular capsules and visible partitions. Partitions and walls were strengthened without wall nodules. Gastritis cystica profunda (GCP): GCP is a rare condition with limited case reports, which is thought to arise from subepithelial migration of epithelial cells particularly following surgical mucosal disruption or due to factors such as ischemia and chronic inflammation.^[19]^ GCP has malignant potential. When gastric cancer arises from the wall of GCP, mural cancerous nodules on the cyst wall will emerge, appearing as mixed cystic and solid lesion that should be differentiated from gastric BCs. On enhanced CT and MRI scans, careful attention should be paid to whether the cyst wall is uniform and whether there are any abnormal enhanced mural nodules in order to differentiate between these 2 conditions. However, the incidence of both GCP and gastric BCs is relatively low with unspecific presentation, rendering accurate preoperative diagnosis challenging. Pathological examination of the resected specimen postoperatively remains the gold standard for definitive diagnosis. On the other hand, BCs with equal or slightly high density on CT should be differentiated from some solid diseases, including low-risk GIST: BC of the stomach can easily be misdiagnosed as GIST, the latter being much more clinically common. GIST are mostly solid, and do not often exhibit necrosis or cystic changes. The cystic changes in GIST tend to be focal with irregular internal surfaces rather than smooth BC and usually do not involve the whole tumor. The proteinaceous content of BC is very helpful in identifying GIST necrosis.^[13]^ Moreover, enhanced scanning is helpful in distinguishing between the 2. GIST usually show mild to moderate delayed enhancement, whereas BC of the stomach shows no enhancement in fluid content. GLM: GLM is more common in the gastric corpus. The tumors were mostly solid masses, with few cystic changes and calcifications. Enhanced scanning is helpful in the differential diagnosis of these 2 diseases. Schwannoma: Schwannomas in the abdominal cavity are mostly located retroperitoneally near the spine. This is typically large when found. CT may reveal a solid mass with uniform density, a mixed-density mass, or a complete cystic lesion. MRI can reveal remote hemorrhage, liquefaction necrosis, collagen fiber components, and edema of the tumor tissue, leading to complex and variable signal characteristics. The heterogeneity of the lesion density and signal and its enhancement features may be used to distinguish it from gastric BCs.

The prognosis of BC in the stomach remains unclear owing to its rarity. Malignant transformation has been reported in 2 cases of gastric adenocarcinoma.^[7,20]^ An increase in serum CEA levels in 1 case was reported, in which a mucinous neoplasm harboring a GNAS gene mutation was observed,^[21]^ suggesting potential malignant transformation. The possible pathology may be chronic inflammation, repeated erosion, and regeneration, which may lead to atypical hyperplasia or cancer.^[7]^ Regarding the indications for surgery, cases with possible malignant transformation, such as those with increasing tumor diameter or elevated serum tumor marker levels, should be treated with surgical resection.^[21]^ In the reported cases, almost all the patients underwent surgical resection. The patient recovered well after surgery and there was no recurrence or metastasis during the follow-up period.

The most regrettable aspect of this case was the lack of clear diagnosis prior to surgery. First, due to the rarity of gastric BC, it is often overlooked and can be challenging to consider during the initial visit. Secondly, the radiographic misdiagnosis of gastric cancer with lymph node metastasis led to the patient undergoing surgery without an attempt to obtain further cytological diagnosis through endoscopic ultrasound-guided fine needle aspiration (EUS-FNA). We emphasize that the possibility of gastric BC should be included in the differential diagnosis when imaging suggests a fluid cyst in the gastric submucosa and enhanced CT indicates a continuous noninvasion of the gastric mucosal surface. EUS-FNA has the potential to detect ciliated respiratory epithelium and mucin on the stomach wall, which may facilitate a definitive diagnosis.

## 4. Conclusions

In conclusion, BC of the stomach is a rare benign lesion. Gastric BC presents across a diverse age spectrum. The clinical symptoms and findings from physical examinations are often nonspecific. Most patients exhibit no abnormalities in laboratory tests; however, some patients display elevated tumor markers, which subsequently normalize following surgical intervention. The majority of gastric BCs are situated in the upper portion of the stomach, predominantly on the lesser curvature side. Typically, the lesions are solitary and vary in size. Imaging manifestations are mostly ovate in shape, with well-defined margins and clear boundaries with adjacent structures, and cyst walls manifest prolonged enhancement. Calcification may occur at the edges. The cyst contents mostly present as low density on CT, hypointensity on T_1_WI, and hyperintensity on T_2_WI. When the protein content of the cyst fluid was high, CT showed equal or slightly higher density and T_1_WI showed hyperintensity. These signs can be used in the differential diagnosis of BCs from other cystic or solid lesions that occur in stomach. By integrating clinical and imaging data from patients, it is anticipated that accurate diagnoses can be made prior to surgery. This approach enhances the understanding of gastric BC and informs the planning of clinical treatment, potentially allowing for conservative treatment options. However, further research is still needed to validate.

## Acknowledgments

The authors appreciate the patient’s consent to present this case.

## Author contributions

**Conceptualization:** Deshuo Dong, Anliang Chen.

**Data curation:** Deshuo Dong.

**Formal analysis:** Anliang Chen.

**Investigation:** Anliang Chen.

**Methodology:** Deshuo Dong.

**Software:** Deshuo Dong, Anliang Chen.

**Writing – original draft:** Deshuo Dong, Anliang Chen.

**Writing – review & editing:** Ailian Liu.
